# Impeller: a path-based heterogeneous graph learning method for spatial transcriptomic data imputation

**DOI:** 10.1093/bioinformatics/btae339

**Published:** 2024-05-28

**Authors:** Ziheng Duan, Dylan Riffle, Ren Li, Junhao Liu, Martin Renqiang Min, Jing Zhang

**Affiliations:** Department of Computer Science, University of California, Irvine, Irvine, CA 92697, United States; Department of Computer Science, University of California, Irvine, Irvine, CA 92697, United States; Mathematical, Computational, and Systems Biology, University of California, Irvine, Irvine, CA 92697, United States; Department of Computer Science, University of California, Irvine, Irvine, CA 92697, United States; Department of Machine Learning, NEC Labs America, Princeton, NJ 08540, United States; Department of Computer Science, University of California, Irvine, Irvine, CA 92697, United States

## Abstract

**Motivation:**

Recent advances in spatial transcriptomics allow spatially resolved gene expression measurements with cellular or even sub-cellular resolution, directly characterizing the complex spatiotemporal gene expression landscape and cell-to-cell interactions in their native microenvironments. Due to technology limitations, most spatial transcriptomic technologies still yield incomplete expression measurements with excessive missing values. Therefore, gene imputation is critical to filling in missing data, enhancing resolution, and improving overall interpretability. However, existing methods either require additional matched single-cell RNA-seq data, which is rarely available, or ignore spatial proximity or expression similarity information.

**Results:**

To address these issues, we introduce Impeller, a path-based heterogeneous graph learning method for spatial transcriptomic data imputation. Impeller has two unique characteristics distinct from existing approaches. First, it builds a heterogeneous graph with two types of edges representing spatial proximity and expression similarity. Therefore, Impeller can simultaneously model smooth gene expression changes across spatial dimensions and capture similar gene expression signatures of faraway cells from the same type. Moreover, Impeller incorporates both short- and long-range cell-to-cell interactions (e.g. via paracrine and endocrine) by stacking multiple GNN layers. We use a learnable path operator in Impeller to avoid the over-smoothing issue of the traditional Laplacian matrices. Extensive experiments on diverse datasets from three popular platforms and two species demonstrate the superiority of Impeller over various state-of-the-art imputation methods.

**Availability and implementation:**

The code and preprocessed data used in this study are available at https://github.com/aicb-ZhangLabs/Impeller and https://zenodo.org/records/11212604.

## 1 Introduction

The orchestration of cellular life hinges on the precise control of when and where genes are activated or silenced. Characterizing such spatiotemporal gene expression patterns is crucial for a better understanding of life, from development to disease to adaptation (Mantri *et al.* 2021). While single-cell RNA sequencing (scRNA-seq) is a revolutionary and widely available technology that enables simultaneous gene expression profiling over thousands of cells, it usually needs to dissociate cells from their native tissue and thus loses the spatial context (Lähnemann *et al.* 2020). Recent advances in spatial transcriptomics (Ståhl *et al.* 2016) allow spatially resolved gene expression measurements at a single-cell or even sub-cellular resolution, providing unprecedented opportunities to characterize the complex landscape of spatiotemporal gene expression and understand the intricate interplay between cells in their native microenvironments (Strell *et al.* 2019). However, due to technical and biological limitations, most spatial transcriptomic profiling technologies still yield incomplete datasets with excessive missing gene expression values, hindering our biological interpretation of such valuable datasets (Choe *et al.* 2023). Therefore, gene imputation is a critical task to enrich spatial transcriptomics by filling in missing data, enhancing resolution, and improving the overall quality and interpretability of the datasets.

Several methods have been successfully developed for gene imputation in spatial transcriptomics, which can be broadly summarized into two categories—reference-based and reference-free approaches. Since scRNA-seq data usually offer a deeper dive into transcriptome profiling, reference-based methods integrated spatial transcriptomic data with matched scRNA-seq data from the same sample for accurate imputation. While promising, these referenced-based methods usually suffer from two limitations. First, most studies do not always have matched scRNA-seq data, especially those using valuable and rare samples. Second, even with matched data, there can be significant gene expression distribution shifts due to sequencing protocol differences (e.g. single nuclei RNA-seq versus whole cell spatial transcriptomics) (Zeng *et al.* 2022).

Researchers also used reference-free methods for direct gene expression imputation. For instance, traditional gene imputation methods designed for scRNA-seq data, such as scVI (Lopez *et al.* 2018), ALRA (Linderman *et al.* 2018), Magic (van Dijk *et al.* 2018), and scGNN (Wang *et al.* 2021), have been adapted for spatial transcriptomic data imputation. While effectively capturing cell-type-specific gene expression signatures, these methods completely ignored the rich spatial information, resulting in suboptimal results. Later, scientists emphasized the importance of spatial context for cell-to-cell interaction (CCI) in modulating expression changes in response to external stimuli (Armingol *et al.* 2021). Therefore, Graph Neural Network (GNN) based methods have been developed to mimic CCIs for imputation tasks with improved performance. However, different types of CCI involve distinct cell signaling mechanisms with varying interaction ranges. Existing GNN-based methods used very shallow convolutional layers for computational convenience, successfully modeling short-range CCI (e.g. via autocrine and juxtacrine) but ignoring long-range interactions (e.g. via paracrine and endocrine). As a result, they cannot fully exploit the spatial information for gene expression imputation.

To address the abovementioned issues, we propose Impeller, a path-based heterogeneous graph learning method for accurate spatial transcriptomic data imputation. Impeller contains two unique components to exploit both transcriptomic and spatial information. First, it builds a heterogeneous graph with nodes representing cells and two types of edges describing expression similarity and spatial proximity. Therefore, the expression-based edges allow it to capture cell-type-specific expression signatures of faraway cells from the same type, and the proximity-based edges incorporate CCI effects in the spatial context. Second, Impeller models long-range CCI by stacking multiple GNN layers and uses a learnable path operator instead of the traditional Laplacian matrices to avoid the over-smoothing problem. Extensive experiments on diverse datasets from three popular platforms and two species demonstrate the superiority of Impeller over various state-of-the-art imputation methods.

Our main contributions are summarized below:

- We propose a graph neural network, Impeller, for reference-free spatial transcriptomic data imputation. Impeller incorporates cell-type-specific expression signatures and CCI via a heterogeneous graph with edges representing transcriptomic similarity and spatial proximity.
- Impeller stacks multiple GNN layers to include both short- and long-range cell-to-cell interactions in the spatial context. Moreover, it uses a learnable path-based operator to avoid over-smoothing.
- To the best of our knowledge, this is the first paper to combine cell-type-specific expression signatures with spatial short- and long-range CCI for gene expression imputation.
- We extensively evaluate Impeller alongside state-of-the-art competitive methods on datasets from three sequencing platforms and two species. The results demonstrate that Impeller outperforms all of the baselines.

## 2 Related work

### 2.1 Imputation methods ignoring spatial information

Earlier spatial transcriptomic data imputation methods adapted the computational strategies originally developed for scRNA-seq data, overlooking the spatial coordinate information of each spot. For instance, eKNN (expression-based K nearest neighbor), and eSNN (expression-based Shared nearest neighbor) are methods implemented using the Seurat R-package that rely on gene expressions of nearest neighbors. MAGIC adopted data diffusion across similar cells to impute missing transcriptomic data. ALARA used low-rank approximation to distinguish genuine nonexpression from technical dropouts, thus preserving true gene absence in samples. scVI used a deep variational autoencoder for gene imputation by assuming the read counts per gene follow a zero-inflated negative binomial distribution. However, these methods completely ignored the rich spatial information, resulting in sub-optimal performance.

### 2.1 Imputation methods utilizing spatial information

Later on, several methods were developed to exploit the spatial coordinate information to improve imputation accuracy. Since scRNA-seq data are usually sequenced deeper to provide more accurate expression measurements, several methods incorporated additional scRNA-seq data during the imputation process. For instance, gimVI used a low-rank approximation and included scRNA reference (Lopez et al. 2019). Tangram mapped scRNA-seq data onto spatial transcriptomics data to facilitate imputation by fitting expression values on the shared genes (Biancalani *et al.* 2021). STLearn used gene expression data, spatial distance, and tissue morphology data for imputing absent gene reads (Pham *et al.* 2020). However, additional scRNA-seq data are not always available and there can be large gene expression distribution shifts between these datasets due to differences in sequencing protocols (e.g. single-cell versus single-nuclei), resulting in limited applications for reference-based methods.

On the other hand, several reference-free methods have been developed for more generalized settings. For example, the seKNN (spatial-expression-based K nearest neighbor) and seSNN (spatial-expression-based shared nearest neighbor) models (Satija *et al.* 2015, Butler *et al.* 2018, Stuart *et al.* 2019, Hao *et al.* 2021) incorporated cell-to-cell distance when defining the KNN for imputation tasks. Recently, STAGATE (Dong and Zhang 2022) is a graph attention auto-encoder framework that effectively imputes genes by integrating spatial data and cell type labels. Overall, these methods did not deeply integrate and exploit the full potential of combining expression and spatial data.

## 3 Materials and methods

### 3.1 Problem definition

Here, we aim to impute the excessive missing gene expression values in spatial transcriptomics data without matched reference scRNA-seq data. Formally, given a sparse cell-by-gene count matrix $X_{\mathrm{obs}}\in R^{n\times m}$ which represents observations for *n* cells across *m* genes, and the spatial coordinates $C\in R^{n\times 2}$ of these cells, our goal is to impute the gene expression matrix $\hat{X}\in R^{n\times m}$. $X_{\mathrm{obs}}$ is derived from the ground truth matrix $X_{\mathrm{gt}}\in R^{n\times m}$, which contains the observed nonzero entries pre-masking. To simulate real-world data conditions, 10% of the nonzero entries in $X_{\mathrm{gt}}$ are masked to form a test set and another 10% for validation, thus creating $X_{\mathrm{obs}}$. This matrix serves as the input for our imputation model. The major challenge is to generate $\hat{X}$ that is as close as possible to the ground truth gene expression $X_{\mathrm{gt}}$, using both the observed gene expressions in $X_{\mathrm{obs}}$ and the spatial information in C.

### 3.2 Heterogeneous graph construction

**Figure 1.**
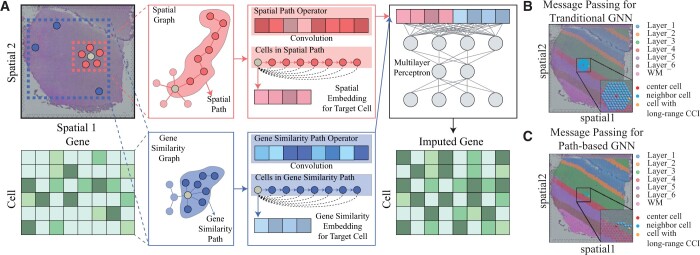
The overview of Impeller. (A) Given the observed matrix $X_{\mathrm{obs}}\in R^{n\times m}$ of *n* cells and *m* genes, and the cells’ spatial coordinates $C\in R^{n\times 2}$, we build the spatial graph $G_{s}$ and the gene similarity graph $G_{g}$. The learned spatial and gene similarity path operators $op_{s}$ and $op_{g}$ are obtained through $\mathrm{pat}h_{s}$ and $\mathrm{pat}h_{g}$, respectively. Convoluting cell features with path operators yields spatial/gene similarity embeddings, which are concatenated and fed into a multilayer perceptron for final gene imputation. (B) and (C) Comparison of neighbor aggregation methods in GNNs. (B) Traditional GNN stacks multiple layers to gather information from distant nodes. (C) The path-based GNN, Impeller samples a path to the target node.

As shown in Fig. 1, we build our Impeller model based on two widely accepted biological insights–(i) gene expression can be modulated by surrounding cells via CCI; (ii) faraway cells of the same cell type may share stable gene expression signatures. Therefore, Impeller first builds a heterogeneous graph **G** to fully exploit both spatial and cell-type information, with nodes and edges representing cells and their relationships.

Specifically, **G** contains two complementary graphs: a spatial graph ($G_{s}$) and a gene similarity graph ($G_{g}$). Edges in $G_{s}$ represent the cell’s spatial proximity to model CCI, while edges in $G_{g}$ denote the cell’s transcriptomic similarity to capture the cell-type-specific expression signatures.

#### 3.2.1 Spatial graph construction

The spatial graph $G_{s}(V_{s},E_{s})$ is created based on the spatial distance between cells, with nodes $V_{s}$ representing the cells and edges in $E_{s}$ connecting nearby cells. Specifically, an edge $e_{s,\{ij\}}$ in $G_{s}$ is established between $v_{i},v_{j}\in V_{s}$ if and only if their Euclidean distance $d_{i,j}$ is less than a predefined threshold $d_{\text{thr}}$, which can be represented as:

(1) $$e_{s,\{ij\}}=\text{if}\;||C_{i}-C_{j}|{|}_{2}\leq d_{\text{thr}}\;\text{else}\;0,$$

where $C_{i}=[C_{i,0},C_{i,1}]$ and $C_{j}=[C_{j,0},C_{j,1}]$ are 2D spatial coordinates of cell *i* and *j*, respectively.

#### 3.2.2 Gene similarity graph construction

Impeller also builds a gene expression similarity graph $G_{g}$ similar to that in scRNA-seq analysis. Specifically, we first extract the highly variable genes (default 3100). Then, for each target cell, we select its top *K* most similar cells. Mathematically,

(2) $$e_{g,\{ij\}}=1\;\text{if}\;j\in K_{g}(X_{i}^{h})\;\text{else}\;0,$$

where $X_{i}^{h}$ is the expression vector of highly variable genes in cell *i*, $K_{g}(X_{i}^{h})$ returns the top *k_g_* cells most similar to cell *i* (e.g. using the Euclidean distance as the similarity metric), and $e_{g,\{ij\}}$ is the edge between cells *i* and *j* in $G_{g}$.

### 3.3 GNN model on heterogeneous graph

With the heterogeneous graph built, Impeller uses a path-based heterogeneous GNN to synthesize the impacts of spatial CCI ($G_{s}$) and cell-type-specific expression signatures ($G_{g}$) for the imputation task. We introduce the problem of traditional GNN, our learnable path operator, and the overall architecture of Impeller as follows.

#### 3.3.1 Problem of traditional GNN

We aim to impute the missing gene expression values in spatial transcriptomics data by incorporating its physical and transcriptional neighbors via a heterogeneous graph. By treating expression profiles as initial cell embeddings ($f^{\left(0\right)}=X_{\mathrm{obs}}$), the *l*th (l∈{1,2,…,L−1}) GNN layer follows a message passing form (Duan *et al.* 2022a^,^^b^^,^^c^, Wang *et al.* 2019, 2022, Xu *et al.* 2020, 2021, Duan *et al.* 2023, 2024) to generate cell *i’*s embedding in layer *l* as follows:

(3) $$\begin{array}{c} f_{i}^{\left(l\right)}=\gamma_{\Theta }(f_{i}^{\left(l-1\right)}\underset{j\in N_{s}(i)}{⊕}\phi_{\Theta }(f_{i}^{\left(l-1\right)},f_{j}^{\left(l-1\right)},e_{s,\{ij}\})\\ \underset{j\in N_{g}(i)}{⊕}\psi_{\Theta }(f_{i}^{\left(l-1\right)},f_{j}^{\left(l-1\right)},e_{g,\{ij}\})), \end{array}$$

where $f_{i}^{\left(l\right)}\in R^{d_{\mathrm{emb}}^{\left(l\right)}}$ is the embedding of cell *i* at *l*th layer, $d_{\mathrm{emb}}^{\left(l\right)}$ is the embedding dimension at *l*th layer, and $N_{s}(i)$ and $N_{g}(i)$ are neighboring cell *i* in $G_{s}$ and $G_{g}$. ⊕ denotes a differentiable, permutation invariant function, e.g. sum, mean, and $\gamma_{\Theta },\;\phi_{\Theta }$, and $\psi_{\Theta }$ denote differentiable functions such as MLPs. After *L* layers, we obtain the imputed gene expressions, denoted as $\hat{X}=f^{\left(L\right)}\in R^{n\times m}$.

In order to capture long-range CCI interaction, we have to include relatively far away cells by stacking multiple GNN layers via a larger *L*. Traditional Laplacian matrices-based GNN suffers from over-smoothing, resulting in deteriorated performance as *L* increases (Eliasof *et al.* 2022). Therefore, we introduce a learnable path operator to overcome this issue and better capture the long-range CCI.

#### 3.3.2 Learnable path operator

We first define path $P_{s}=(s_{1},s_{2},\ldots ,s_{k_{s}})$ on $G_{s}$ of length *k_s_* and path $P_{g}=(g_{1},g_{2},\ldots ,g_{k_{g}})$ on $G_{g}$ of length *k_g_*, where *s_i_* and *g_i_* are node (cell) indexes. Node embeddings at *l*th layer are denoted by $f_{s_{i}}^{\left(l\right)}\in R^{d_{\mathrm{emb}}^{\left(l\right)}}$ and $f_{g_{i}}^{\left(l\right)}\in R^{d_{\mathrm{emb}}^{\left(l\right)}}$. Then, $op_{s}^{\left(l\right)}\in R^{k_{s}\times d_{\mathrm{emb}}^{\left(l\right)}}$ and $op_{g}^{\left(l\right)}\in R^{k_{g}\times d_{\mathrm{emb}}^{\left(l\right)}}$ are two learnable path operators which allow us to convolve node embeddings along paths:

(4) $$\begin{array}{cc} op_{s}^{\left(l\right)}(P_{s})*f^{\left(l\right)} & =\sum_{i=1}^{k_{s}}op_{s,i}^{\left(l\right)}*f_{s_{i}}^{\left(l\right)}=\sum_{i=1}^{k_{s}}\sum_{j=1}^{d_{\mathrm{emb}}^{\left(l\right)}}op_{s,i}^{\left(l\right)}[j]\cdot f_{s_{i}}^{\left(l\right)}[j],\\ op_{g}^{\left(l\right)}(P_{g})*f^{\left(l\right)} & =\sum_{i=1}^{k_{g}}op_{g,i}^{\left(l\right)}*f_{g_{i}}^{\left(l\right)}=\sum_{i=1}^{k_{g}}\sum_{j=1}^{d_{\mathrm{emb}}^{\left(l\right)}}op_{g,i}^{\left(l\right)}[j]\cdot f_{g_{i}}^{\left(l\right)}[j], \end{array}$$

where ′*′ denotes the convolution operation, and ′·′ symbol is the multiplication operation between two scalars. Here $op_{s,i}^{\left(l\right)}[j],\;op_{g,i}^{\left(l\right)}[j],\;f_{s_{i}}^{\left(l\right)}[j]$ and $f_{g_{i}}^{\left(l\right)}[j]$ represent the *j*th scalars of the $d_{\mathrm{emb}}^{\left(l\right)}$-dimensional vector $op_{s,i}^{\left(l\right)},\;op_{g,i}^{\left(l\right)},\;f_{s_{i}}^{\left(l\right)}$ and $f_{g_{i}}^{\left(l\right)}$, respectively. Starting from each node, we generate multiple paths on $G_{s}$ and $G_{g}$ and aggregate results for a more expressive representation:

(5) $$\begin{array}{cc} op_{s}^{\left(l\right)}(P_{s})*f^{\left(l\right)} & =\frac{1}{T_{s}}\sum_{P_{s}\in P_{s}}op_{s}^{\left(l\right)}(P_{s})*f^{\left(l\right)},\\ op_{g}^{\left(l\right)}(P_{g})*f^{\left(l\right)} & =\frac{1}{T_{g}}\sum_{P_{g}\in P_{g}}op_{g}^{\left(l\right)}(P_{g})*f^{\left(l\right)}, \end{array}$$

where $P_{s}$ and $P_{g}$ are sets of paths sampled from the $G_{s}$ and $G_{g}$, each containing *T_s_* and *T_g_* paths. Each path $P_{s}\in P_{s}$ and $P_{g}\in P_{g}$ are separately convolved using $op_{s}^{\left(l\right)}$ or $op_{g}^{\left(l\right)}$, and the results are averaged to acquire the node embeddings.

#### 3.3.3 The overall architecture of impeller

After convolving both spatial and gene similarity paths, we concatenate their embeddings to form the overall node embeddings, as in

(6) $$f^{\left(l+1\right)}=\sigma (W_{1}^{\left(l\right)}[op_{s}^{\left(l\right)}(P_{s})*f^{\left(l\right)},op_{g}^{\left(l\right)}(P_{g})*f^{\left(l\right)}]),$$

where σ(·) denotes the ReLU activation function, $W^{\left(l\right)}\in R^{d_{\mathrm{emb}}^{\left(l+1\right)}\times 2*d_{\mathrm{emb}}^{\left(l\right)}}$ is the learnable weight matrix, $d_{\mathrm{emb}}^{\left(l\right)}$ is the embedding dimension at *l*th layer, and [·,·] denotes concatenation operation. Then, Impeller tries to minimize the Mean Squared Error (MSE) between $\hat{X}$ and $X_{\mathrm{gt}}$:

(7) $$L=\frac{\sum_{i=1}^{n}\sum_{j=1}^{m}\;1[X_{gt,(i,j)}\neq 0]{\left({\hat{X}}_{i,j}-X_{gt,(i,j)}\right)}^{2}}{\sum_{i=1}^{n}\sum_{j=1}^{m}\;1[X_{gt,(i,j)}\neq 0]},$$

where 1[·] is an indicator function that equals 1 if the condition inside brackets is met ($X_{gt,(i,j)}\neq 0$), and 0 otherwise. The loss is computed only over nonzero entries of $X_{\mathrm{gt}}$.

### 3.4 Computational complexity analysis

#### 3.4.1 *k*-hop complexity analysis

Traditional GNNs need to gather information from *k*-hop neighbor nodes after stacking of *k* layers. Given the complexity of each layer as $O(n\times d_{t})$, where *n* is the number of nodes and *d_t_* is the average node degree, the overall complexity becomes $O(n\times d_{t}\times k)$. In contrast, Impeller can directly access neighbors up to *k*-hop distance via a single layer by setting $k_{s}=k_{g}=k$. The computational complexity per layer for Impeller is $O(n\times (T_{s}\times k_{s}+T_{g}\times k_{g}))$, with *T_s_* and *T_g_* representing the number of paths in $G_{s}$ and $G_{g}$, *k_s_* and *k_g_* denoting path lengths. As a result, when $T_{s}<d_{t}$ (a condition satisfied in our task), Impeller offers superior computational efficiency.

#### 3.4.2 The number of parameters

Traditional GNNs have $O(d_{\mathrm{emb}}^{\left(l\right)}\times d_{\mathrm{emb}}^{\left(l+1\right)})$ parameters per layer while the path operator of Impeller adds $(k_{s}+k_{g})*d_{\mathrm{emb}}^{\left(l\right)}$ parameters. Since $k_{s}+k_{g}$ is typically much smaller than $d_{\mathrm{emb}}^{\left(l+1\right)}$, Impeller’s number of parameters remains on par with traditional GNNs.

## 4 Experiments

### 4.1 Detailed experimental setup

#### 4.1.1 Data sources and preprocessing

In our study, we tested Impeller using diverse datasets from three popular sequencing platforms and two organisms. Specifically, we included 10X Visium datasets from the human dorsolateral prefrontal cortex (DLPFC), (Maynard *et al.* 2021), Steroseq datasets from mouse olfactory bulb (Chen *et al.* 2021), and Slide-seqV2 from mouse olfactory bulb (Stickels *et al.* 2021) in our analyses. Detailed attributes of these datasets are summarized in Table 1 (for filter details and visualizations, see the Supplementary Appendix). After standard pre-processing and normalization procedures, we downsampled the data according to scGNN, where 10% of nonzero entries in the dataset were used as a test set, and another 10% of nonzero entries were reserved for validation. For a fair comparison, we repeat ten times with different mask configurations.

**Table 1.** Summary of datasets.

Platform	Organism	Sample ID	Raw matrix (cell, gene)	Raw density	Filter matrix (cell, gene)	Filter density	No. of imputed entries
10xVisium	Human dorsolateral prefrontal cortex (DLPFC)	151507	4226, 33 538	0.042	4117, 4028	0.261	437 240
		151508	4384, 33 538	0.036	4148, 3342	0.258	358 184
		151509	4789, 33 538	0.043	4700, 4188	0.258	508 186
		151510	4643, 33 538	0.041	4547, 3908	0.259	461 112
		151669	3661, 33 538	0.054	3617, 5246	0.277	525 930
		151670	3498, 33 538	0.050	3433, 4909	0.272	457 770
		151671	4110, 33 538	0.055	3988, 5539	0.278	615 111
		151672	4015, 33 538	0.052	3809, 5273	0.279	561 166
		151673	3639, 33 538	0.066	3628, 6538	0.286	677 473
		151674	3673, 33 538	0.080	3668, 7796	0.305	871 032
		151675	3592, 33 538	0.054	3565, 5454	0.267	518 515
		151676	3460, 33 538	0.058	3449, 5784	0.274	545 920
Stereoseq	Mouse	/	19 109, 14 376	0.024	4036, 1581	0.193	123 444
SlideseqV2	Mouse	/	20 139, 11 750	0.031	5161, 2611	0.217	292 418

#### 4.1.2 Baseline methods for benchmarking

We conducted a comparative study utilizing 12 state-of-the-art methods, including reference-free and reference-based methods that originally required additional scRNA-seq data. However, in our analysis, we did not use any additional scRNA-seq data for a fair comparison.

First, we included methods directly adapted from scRNA-seq data imputation and completely ignored the rich spatial information, including a deep generative model scVI, a low-rank approximation model ALRA, nearest neighbors-based models eKNN and eSNN, a diffusion-based model MAGIC, and a GNN-based model scGNN. Furthermore, we used several imputation methods specifically designed for spatial transcriptomic data, such as seKNN (spatial-expression-based K nearest neighbor), and seSNN (spatial-expression-based shared nearest neighbor). gimVI and Tangram need additional scRNA-seq from matched samples, so we used a reference-free implementation available through their website for a fair comparison. Lastly, we included STAGATE a graph attention auto-encoder framework by amalgamating spatial data and gene expression profiles. We use default parameters in most baseline methods (for details, see the Supplementary Appendix).

#### 4.1.3 Evaluation metrics

We first define a test mask $M\in R^{n\times m}$ where the entries to be imputed are marked as 1 and the others as 0. Then we extract the relevant entries from both the imputed matrix $\hat{X}$ and the ground truth matrix $X_{\mathrm{gt}}$ to form two vectors: $\hat{x}$ (from $\hat{X}$) and $x_{\mathrm{gt}}$ (from $X_{\mathrm{gt}}$), each of length *N*, where *N* is the total number of entries to be imputed. Following scGNN settings, we use L1 Distance, Cosine Similarity, and Root-Mean-Square Error (RMSE) to compare imputed gene expressions $\hat{x}$ with the ground truth $x_{\mathrm{gt}}$. Mathematically:

(8) $$L1\;\text{Distance}=|\hat{x}-x_{\mathrm{gt}}|,$$

 

(9) $$\text{Cosine Similarity}(\hat{x},x_{\mathrm{gt}})=\frac{\hat{x}x_{\mathrm{gt}}{\;}^{T}}{\left||\hat{x}||*||x_{\mathrm{gt}}|\right|},$$

 

(10) $$\text{RMSE}(\hat{x},x_{\mathrm{gt}})=\sqrt{\frac{\sum_{i=1}^{N}{\left({\hat{x}}_{i}-x_{\mathrm{gt}}{\;}_{i}\right)}^{2}}{N}}.$$

### 4.2 Experimental results

#### 4.2.1 Improved imputation accuracy

We benchmarked our performance against 12 leading methods by assessing imputation accuracy across 14 datasets. These datasets span three prominent sequencing platforms (10x Visium, Stereoseq, and Slideseq) and two species (human and mouse). Table 2 summarizes the performance of Impellers and other baselines (for results of the other six samples of the DLPFC dataset, please see the Supplementary Appendix). For a fair comparison, we did not include any additional scRNA-seq data to facilitate the imputation task. Overall, Impeller consistently outperforms others in all datasets using L1 distance, Cosine Similarity, and RMSE, indicating the effectiveness and robustness of our strategy.

**Table 2.** Gene imputation benchmark.^a^

Metric	Method		Platform and dataset							
			10xVisium						Stereoseq	SlideseqV2
			DLPFC						Mouse	Mouse
			151507	151508	151509	151510	151669	151670	/	/
L1 distance	wo	scVI	0.794 ± 0.004	0.838 ± 0.006	0.800 ± 0.002	0.670 ± 0.003	0.810 ± 0.003	0.696 ± 0.005	1.442 ± 0.005	1.127 ± 0.006
		ALRA	0.499 ± 0.003	0.512 ± 0.001	0.490 ± 0.001	0.496 ± 0.001	0.467 ± 0.002	0.472 ± 0.002	0.406 ± 0.013	0.649 ± 0.066
		eKNN	0.274 ± 0.001	0.281 ± 0.001	0.275 ± 0.000	0.275 ± 0.001	0.269 ± 0.000	0.272 ± 0.000	0.205 ± 0.001	0.294 ± 0.001
		eSNN	1.254 ± 0.001	1.373 ± 0.001	1.266 ± 0.001	1.294 ± 0.000	1.017 ± 0.001	1.071 ± 0.001	2.802 ± 0.002	2.071 ± 0.002
		Magic	0.779 ± 0.001	0.825 ± 0.001	0.787 ± 0.000	0.664 ± 0.001	0.795 ± 0.001	0.692 ± 0.000	1.324 ± 0.001	1.080 ± 0.000
		scGNN	0.583 ± 0.011	0.665 ± 0.085	0.589 ± 0.011	0.584 ± 0.004	0.550 ± 0.006	0.532 ± 0.009	0.819 ± 0.240	0.664 ± 0.018
	w	gimVI	0.838 ± 0.003	0.890 ± 0.003	0.835 ± 0.001	0.737 ± 0.002	0.863 ± 0.003	0.765 ± 0.001	1.325 ± 0.001	1.153 ± 0.002
		seKNN	0.306 ± 0.000	0.309 ± 0.001	0.307 ± 0.000	0.307 ± 0.000	0.281 ± 0.000	0.289 ± 0.000	0.263 ± 0.001	0.876 ± 0.001
		seSNN	1.254 ± 0.001	1.371 ± 0.001	1.266 ± 0.000	1.294 ± 0.000	1.017 ± 0.001	1.072 ± 0.001	2.775 ± 0.002	1.998 ± 0.001
		Tangram	1.691 ± 0.001	1.811 ± 0.001	1.689 ± 0.000	1.420 ± 0.000	1.728 ± 0.001	1.474 ± 0.000	2.899 ± 0.001	2.185 ± 0.000
		STLearn	1.333 ± 0.001	1.423 ± 0.001	1.332 ± 0.001	1.148 ± 0.001	1.369 ± 0.002	1.206 ± 0.001	NA	NA
		STAGATE	0.297 ± 0.001	0.300 ± 0.002	0.295 ± 0.005	0.294 ± 0.004	0.274 ± 0.005	0.278 ± 0.002	0.289 ± 0.006	0.502 ± 0.007
		Impeller	**0.248 ± 0.001**	**0.252 ± 0.003**	**0.247 ± 0.003**	**0.254 ± 0.003**	**0.242 ± 0.004**	**0.237 ± 0.001**	**0.190 ± 0.005**	**0.292 ± 0.005**
Cosine similarity	wo	scVI	0.907 ± 0.001	0.913 ± 0.001	0.906 ± 0.001	0.903 ± 0.001	0.909 ± 0.001	0.904 ± 0.001	0.941 ± 0.001	0.919 ± 0.002
		ALRA	0.948 ± 0.002	0.952 ± 0.002	0.952 ± 0.001	0.952 ± 0.001	0.938 ± 0.006	0.944 ± 0.003	0.980 ± 0.002	0.927 ± 0.018
		eKNN	0.983 ± 0.000	0.984 ± 0.000	0.983 ± 0.000	0.984 ± 0.000	0.979 ± 0.000	0.979 ± 0.000	0.993 ± 0.000	0.989 ± 0.000
		eSNN	0.842 ± 0.000	0.841 ± 0.000	0.839 ± 0.000	0.840 ± 0.000	0.846 ± 0.000	0.843 ± 0.000	0.777 ± 0.001	0.838 ± 0.000
		Magic	0.915 ± 0.000	0.920 ± 0.000	0.914 ± 0.000	0.909 ± 0.000	0.916 ± 0.000	0.910 ± 0.000	0.968 ± 0.002	0.936 ± 0.000
		scGNN	0.933 ± 0.004	0.927 ± 0.016	0.932 ± 0.002	0.936 ± 0.000	0.917 ± 0.002	0.929 ± 0.002	0.948 ± 0.035	0.953 ± 0.002
	w	gimVI	0.957 ± 0.000	0.965 ± 0.001	0.955 ± 0.001	0.947 ± 0.001	0.962 ± 0.001	0.948 ± 0.002	0.964 ± 0.000	0.936 ± 0.001
		seKNN	0.982 ± 0.000	0.985 ± 0.000	0.982 ± 0.000	0.983 ± 0.000	0.979 ± 0.000	0.980 ± 0.000	0.995 ± 0.000	0.982 ± 0.000
		seSNN	0.843 ± 0.000	0.841 ± 0.000	0.840 ± 0.000	0.841 ± 0.000	0.851 ± 0.000	0.847 ± 0.000	0.768 ± 0.000	0.817 ± 0.000
		Tangram	0.713 ± 0.001	0.725 ± 0.001	0.717 ± 0.001	0.716 ± 0.001	0.717 ± 0.001	0.715 ± 0.000	0.772 ± 0.001	0.763 ± 0.001
		STLearn	0.718 ± 0.000	0.718 ± 0.000	0.715 ± 0.001	0.724 ± 0.000	0.715 ± 0.001	0.717 ± 0.000	NA	NA
		STAGATE	0.983 ± 0.000	0.985 ± 0.000	0.983 ± 0.001	0.984 ± 0.001	0.980 ± 0.001	0.980 ± 0.000	0.990 ± 0.000	0.961 ± 0.000
		Impeller	**0.987 ± 0.000**	**0.988 ± 0.000**	**0.987 ± 0.000**	**0.987 ± 0.000**	**0.983 ± 0.001**	**0.985 ± 0.000**	**0.997 ± 0.000**	**0.990 ± 0.000**
RMSE	wo	scVI	0.940 ± 0.005	0.993 ± 0.006	0.949 ± 0.003	0.803 ± 0.003	0.959 ± 0.003	0.834 ± 0.005	1.628 ± 0.005	1.307 ± 0.007
		ALRA	0.784 ± 0.003	0.810 ± 0.005	0.766 ± 0.001	0.777 ± 0.001	0.735 ± 0.004	0.743 ± 0.003	0.723 ± 0.036	1.061 ± 0.107
		eKNN	0.380 ± 0.001	0.395 ± 0.002	0.382 ± 0.001	0.384 ± 0.001	0.368 ± 0.001	0.374 ± 0.001	0.402 ± 0.008	0.416 ± 0.003
		eSNN	1.378 ± 0.001	1.503 ± 0.000	1.393 ± 0.000	1.419 ± 0.001	1.143 ± 0.002	1.199 ± 0.001	2.778 ± 0.001	2.177 ± 0.001
		Magic	0.917 ± 0.001	0.972 ± 0.001	0.929 ± 0.000	0.792 ± 0.000	0.936 ± 0.001	0.824 ± 0.000	1.453 ± 0.001	1.238 ± 0.001
		scGNN	0.755 ± 0.016	0.850 ± 0.096	0.762 ± 0.011	0.755 ± 0.002	0.717 ± 0.007	0.686 ± 0.010	1.051 ± 0.307	0.842 ± 0.021
	w	gimVI	0.955 ± 0.002	1.002 ± 0.001	0.957 ± 0.001	0.858 ± 0.001	0.970 ± 0.002	0.890 ± 0.002	1.448 ± 0.001	1.217 ± 0.004
		seKNN	0.392 ± 0.001	0.395 ± 0.000	0.392 ± 0.000	0.392 ± 0.000	0.361 ± 0.001	0.370 ± 0.000	0.361 ± 0.001	0.523 ± 0.000
		seSNN	1.354 ± 0.001	1.474 ± 0.000	1.370 ± 0.000	1.395 ± 0.001	1.119 ± 0.001	1.175 ± 0.001	2.770 ± 0.002	2.087 ± 0.001
		Tangram	1.768 ± 0.001	1.889 ± 0.001	1.767 ± 0.000	1.503 ± 0.000	1.804 ± 0.001	1.557 ± 0.000	2.970 ± 0.001	2.284 ± 0.000
		STLearn	1.516 ± 0.001	1.629 ± 0.001	1.521 ± 0.001	1.300 ± 0.001	1.556 ± 0.002	1.362 ± 0.001	NA	NA
		STAGATE	0.384 ± 0.002	0.393 ± 0.002	0.379 ± 0.007	0.380 ± 0.007	0.357 ± 0.007	0.365 ± 0.004	0.485 ± 0.008	0.765 ± 0.005
		Impeller	**0.337 ± 0.001**	**0.341 ± 0.000**	**0.336 ± 0.000**	**0.340 ± 0.004**	**0.327 ± 0.007**	**0.323 ± 0.000**	**0.277 ± 0.002**	**0.391 ± 0.006**

a The best results are bolded. Results marked “NA” for stLearn indicate unavailable HE stained images required by the method.

In addition, we found that most methods utilizing spatial information (w* group in Table 2) demonstrated higher imputation accuracy than those ignoring spatial information (wo* group in Table 2), validating the presence of rich information in the spatial context. Notably, Impeller surpasses even the best gene expression-only method, eKNN, with improvements of 11.32% on 10xVisium DLPFC, 31.09% on Stereoseq, and 6.01% on SlideseqV2 Mouse. Furthermore, compared to uniform averaging using KNN, GNN allows for more flexible neighbor information aggregation for better imputation accuracy, as reflected by the noticeably improved performance of Impeller and STAGATE.

#### 4.2.2 Impact of long-range CCI

To probe disparities between Impeller and traditional GNNs in capturing long-range cell dependencies, we examined several models–Impeller, GCN (Kipf and Welling 2016), GraphSAGE (Hamilton *et al.* 2017), GAT (Veličković *et al.* 2017), and GraphTransformer (Shi *et al.* 2020)–across varying receptive fields in the Stereoseq dataset.

In Table 3, GAT and GraphSAGE suffer from gradient vanishing/exploding issues as more layers are added to capture long-range CCI, resulting in quickly degraded performance. GCN works best initially, but its performance drops with more layers added. This could be because the number of neighbors grows fast as we increase the receptive field, leaving it difficult for the target cell to understand the influence of each neighbor. Furthermore, GraphTransformer starts with high errors at a receptive field of 2. It works best at a receptive field of 8, but the error goes up again at 32. This increase in error is similar to the problem of GCN, as all cells start to look too similar to make useful representations. On the other hand, Impeller effectively tackles these challenges by the path operator, as reflected by the consistently improved results until the receptive field of 32. As the receptive field continues to grow, Impeller’s performance slightly declines, likely because distant information becomes less relevant for the target cell’s gene imputation. An additional perturbation study, demonstrating the effectiveness of Impeller in capturing CCI, is shown in the Supplementary Appendix.

**Table 3.** Performance of different receptive fields (RMSE).

Receptive field	GCN	GraphSAGE	GAT	GraphTransformer	Impeller
2	0.339 ± 0.000	0.352 ± 0.008	0.360 ± 0.005	1.058 ± 0.463	0.310±0.016
4	0.348 ± 0.001	0.362 ± 0.013	0.372 ± 0.022	0.424 ± 0.031	0.286±0.009
8	0.386 ± 0.012	0.496 ± 0.033	0.454 ± 0.024	0.351 ± 0.002	0.279±0.001
16	0.403 ± 0.001	0.617 ± 0.054	0.506 ± 0.061	0.435 ± 0.017	0.286±0.010
32	0.418 ± 0.015	1.466 ± 0.031	1.458 ± 0.037	0.420 ± 0.000	0.277±0.002
64	0.430 ± 0.002	1.615 ± 0.010	1.621 ± 0.004	0.420 ± 0.001	0.302±0.028
128	0.429 ± 0.001	1.629 ± 0.003	1.614 ± 0.022	0.420 ± 0.001	0.357±0.001

The best imputation performance is highlighted in bold.

#### 4.2.3 Advantage of heterogeneous graph

**Figure 2.**
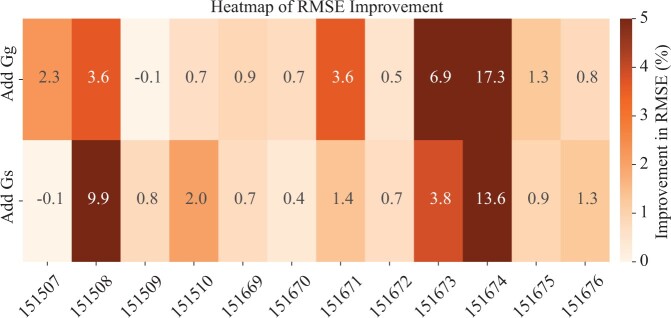
RMSE improvement by adding different graph modalities.

In our study, we explored the influence of graph modalities on imputation accuracy by assessing three key variants: *var_s_*, using solely the spatial graph; *var_g_*, utilizing only the gene similarity graph; *var_h_*, integrating both graphs. We then calculated the performance improvement from adding $G_{g}$ by comparing *var_h_* with *var_s_*, and the improvement from adding $G_{s}$ by comparing *var_h_* with *var_g_*. As shown in Fig. 2, the majority of the cases (22 out of 24) exhibit positive improvements. Specifically, in the DLPFC sample 151674, the inclusion of the gene similarity graph yields a 17.3% improvement, and the 13.6% enhancement is achieved by adding the spatial graph alone. Similarly, in sample 151508, the gene similarity graph and the spatial graph contribute to improvements of 3.6% and 9.9%, respectively. These results underscore the efficacy of our approach, particularly in scenarios where the complex interaction between spatial and gene expression data is pivotal for enhancing gene imputation accuracy.

### 4.3 Ablation study

**Figure 3.**
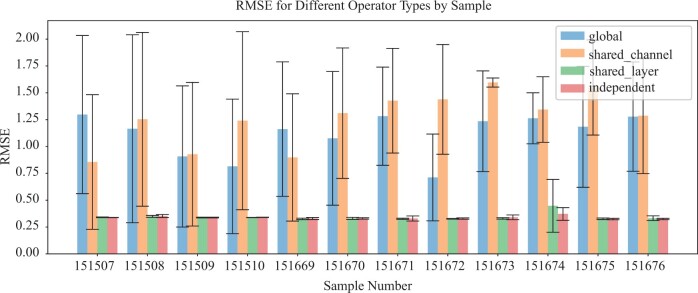
RMSE w.r.t. different path operators.

We conducted an ablation study to evaluate the performance of four primary path operator variants of Impeller: $op_{\mathrm{glo}}$, where all Impeller layers and channels (each channel representing 1D of $f^{\left(l\right)}$) share one path operator; $op_{\mathrm{cha}}$, where channels share an operator but layers have distinct ones; $op_{\mathrm{lay}}$, where all layers share one, but channels have individual operators; and $op_{\mathrm{ind}}$ where every layer and channel possesses an independent path operator. As depicted in Fig. 3, both $op_{\mathrm{glo}}$ and $op_{\mathrm{cha}}$ performed poorly on the DLPFC dataset, indicating the importance of distinct operators for each channel. Notably, $op_{\mathrm{lay}}$ and $op_{\mathrm{ind}}$ showed comparable results, suggesting that layer-specific operators might be optional, depending on the specific application. Another ablation study regarding different path construction and graph construction methods is shown in the Supplementary Appendix.

### 4.4 Parameter analysis

To investigate the influence of Impeller’s various hyper-parameters, we conducted extensive experiments using the DLPFC dataset (Sample ID: 151507) and reported the mean and standard deviation of the imputation accuracy over ten repetitions.

**Figure 4.**
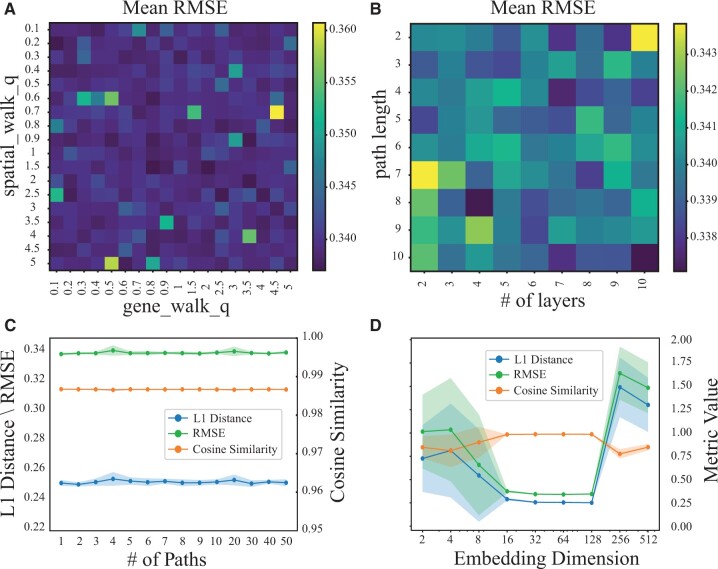
Parameter analysis. (A) The mean RMSE w.r.t. different *q_s_* and *q_g_* for generating random walks in $G_{s}$ and $G_{g}$. (B) The mean RMSE w.r.t. different path lengths *k_s_* and *k_g_*, and the number of Impeller layers *L*. (C) The L1 distance, cosine similarity, and RMSE w.r.t. different number of paths *T_s_* and *T_g_*. (D) The L1 distance, cosine similarity and RMSE w.r.t. different embedding dimensions $d_{\mathrm{emb}}^{\left(l\right)}$.

First, we studied the impact of *q_s_* and *q_g_* on the RMSE of a random walk on $G_{s}$ and $G_{g}$ following the Node2Vec mechanism. Higher values of *q* (i.e. *q_s_* and *q_g_*) encourage the walk to sample more distant nodes, enhancing the exploration of the global graph structure, while lower values bias the walk toward neighboring nodes, facilitating local exploration. As shown in Fig. 4A, Impeller exhibits strong robustness with RMSE from 0.33 to 0.36 when *q_s_* and *q_g_* varied from 0.1 to 5. However, higher values of *q_s_* and *q_g_* tend to induce larger errors. For generality, we selected 1 as the default value for *q_s_* and *q_g_*.

We investigated the impact of random walk length (*k_s_* and *k_g_*) and layer number (*L*), shown in Fig. 4B. A path length of 2 with 10 layers results in maximum errors, reducing our model to a standard ten-layer GCN. This is because, at this path length, the model focuses on immediate neighbors, akin to how traditional GCNs operate. Such a setup, while deep, limits neighborhood exploration and increases over-smoothing risk. Conversely, a path length of 8 with 4 layers allows for capturing broader interactions (up to 28 hops), balancing extended reach and computational efficiency, thus avoiding over-smoothing and optimizing long-range CCI capture.

Next, we examined the impact of the number of random walks (*T_s_* and *T_g_*). As shown in Fig. 4C, *T_s_* and *T_g_* appeared to have a minimal effect on results, due to the robustness of Impeller which resamples at each epoch during training. We chose 8 as the default number of random walks.

Lastly, we evaluated how the embedding dimension $d_{\mathrm{emb}}^{\left(l\right)}$ affects Impeller’s performance. As shown in Fig. 4D, smaller $d_{\mathrm{emb}}^{\left(l\right)}$ (such as 2, 4, 8) leads to limited expressive power and larger imputation errors. As $d_{\mathrm{emb}}^{\left(l\right)}$ increases to 16, 32, 64, or 128, Impeller’s expressive power improves and operations converge well in each run. Due to our early stopping criterion, we cease training if the validation RMSE does not improve for 50 consecutive epochs. When $d_{\mathrm{emb}}^{\left(l\right)}$ was set to 256 or 512, it’s hard for Impeller to converge quickly at these dimensions. To strike a balance between complexity and representational power, we opted for $d_{\mathrm{emb}}^{\left(l\right)}$ of 64.

In summary, these comprehensive parameter analyses reveal that Impeller is robust across a wide range of parameter settings, while still providing tunable options for balancing computational efficiency and prediction accuracy. These results further substantiate the effectiveness and practicality of our proposed model for gene imputation tasks.

### 4.5 Neighbor visualization

To better understand the differences between traditional GNNs and our path-based GNN, Impeller, we turned to a visual example (sample 151507 from the DLPFC dataset). Figure 1B shows how the typical GNN gathers information from far-away neighbors. The center node (red sphere) stacks five GNN layers to gather information from distant nodes like the one shown in yellow. But this method sometimes pulls in extra information from different tissue layers that is not needed. On the other hand, Fig. 1C shows our Impeller model. Instead of stacking GNN layers, Impeller samples a direct path from the center node to the target node. While using this direct path method, Impeller offers better gene imputation performance by capturing the relevant long-range CCI.

### 4.6 Running time analysis

As shown in Table 4, we conducted a comparative model parameter and runtime analysis with popular graph-based models (GCN, GAT, GraphSAGE, and Transformer) on the DLPFC dataset. As discussed in Section 3.4.2, our model maintains a parameter count comparable to traditional GNNs, with the complexity per layer defined as $O(d_{\mathrm{emb}}^{\left(l\right)}\times d_{\mathrm{emb}}^{\left(l+1\right)})$. Specifically, our model introduces only a 3.5% increase in parameters for GCN and a 2.7% increase for GAT. In contrast, it achieves a 48.0% reduction in parameters for GraphSAGE and a 74.1% reduction for GraphTransformer (Table 4). Despite its additional path sampling step, Impeller remarkably outperformed the others in training and inference efficiency. This can be partially credited to leveraging the DGL library’s optimized implementation for path sampling (https://docs.dgl.ai/en/0.8.x/api/python/dgl.sampling.html) and the inherently faster multiplication process used in path-based convolution compared to edge-wise information aggregation in traditional GNNs. In addition, Impeller showed the lowest RMSE, indicating superior prediction accuracy. Hence, Impeller offers a balanced blend of efficiency and precision for spatial transcriptomic data imputation, outperforming other graph-based models.

**Table 4.** Running time summary of graph-based models.

Model	No. of parameters	Path (ms)	Training (ms)	Inference (ms)	RMSE
GCN	519 676		21.73 ± 10.44	21.04 ± 10.84	0.37 ± 0.02
GAT	523 768		23.84 ± 13.25	24.99 ± 11.41	0.36 ± 0.02
GraphSAGE	1 035 260		18.77 ± 11.06	16.97 ± 12.16	0.38 ± 0.01
Transformer	2 078 704		33.94 ± 16.16	38.13 ± 8.71	0.36 ± 0.01
Impeller	538 108	1.61 ± 0.60	**6.35 ± 0.30**	**8.43 ± 0.25**	**0.34 ± 0.00**

Training and inference times with the fastest performance, as well as the best imputation performance (RMSE), are highlighted in bold.

## 5 Conclusion

In this study, we introduced Impeller, a path-based heterogeneous graph learning approach tailored for spatial transcriptomic data imputation. By constructing a heterogeneous graph capturing both spatial proximity and gene expression similarity, Impeller offers a refined representation of cellular landscapes. Further, its integration of multiple GNN layers, coupled with a learnable path operator, ensures comprehensive modeling of both short and long-range cellular interactions while effectively averting over-smoothing issues. Benchmark tests across diverse datasets spanning various platforms and species underscore Impeller’s superior performance compared to state-of-the-art imputation methods. This work not only establishes Impeller’s prowess in spatial transcriptomic imputation but also underscores its potential to model both short- and long-range cell-cell interactions.

## Supplementary Material

btae339_Supplementary_Data

## Data Availability

The code and preprocessed data used in this study are available at https://github.com/aicb-ZhangLabs/Impeller and https://zenodo.org/records/11212604.
