# Supplementary material for: Impeller: a path-based heterogeneous graph learning method for spatial transcriptomic data imputation
Source: Bioinformatics. 2024 May 28;40(6):btae339. doi: 10.1093/bioinformatics/btae339 (PMC11256934; doi:10.1093/bioinformatics/btae339)
Supplement: btae339_Supplementary_Data [file btae339_supplementary_data.pdf]

Appendix for
Impeller: A Path-based Heterogeneous Graph Learning Method for Spatial Transcriptomic Data Imputation

Ziheng Duan[1], Dylan Riffle[1], Ren Li[2], Junhao Liu[1], Martin Renqiang Min[3], and Jing Zhang[1]

[1] Department of Computer Science, University of California, Irvine, CA 92697, USA
[2] Mathematical, Computational, and Systems Biology, University of California, Irvine, Irvine, CA 92697, USA
[3] NEC Labs America, Princeton, NJ 08540, USA

1 Data Preprocessing Details

We included 10xVisum datasets from human Dorsolateral Prefrontal Cortex (DLPFC) [10], Steroseq datasets from mouse olfactory bulb [3], and Slide-seqV2 from mouse olfactory bulb [14] in our analyses. For data preprocessing, we first filtered the cells and genes for quality assurance. Only cells with at least 500 detected genes and genes expressed in at least 10% of cells were retained. Next, we normalized the total counts per cell to a target sum of 1e4 and applied a log transformation to the data. These preprocessing steps aimed to standardize the scale of gene expressions and stabilize the variance, rendering the data more amenable for the subsequent imputation process. The visualizations of this filtering process are shown in **Fig S1**, **Fig S2** and **Fig S3**.

2 Additional Imputation Performance

The imputation results of the other six samples from DLPFC are shown in **Table 1**. Impeller consistently achieves the best performance.

3 Implementation Details

We use the default parameters as suggested in most baseline methods (details see the supplementary material). For implementation details, we built $\mathbf{G_s}$ with d_{thr} at 150. We constructed the $\mathbf{G_g}$ by designating the 5 nearest neighbors. The path length parameters, k_s and k_g, and the number of paths parameters, T_s and T_g for both the spatial and gene similarity graphs were fixed at 8. The parameters for Node2Vec [6] like random walks, p_s, p_g, q_s, and q_g, were uniformly set to 1. In terms of model architecture, we set the number of layers, L, to four and selected an embedding dimension, $d_{emb}^{(l)}$, of 64. We employed the Adam optimizer with

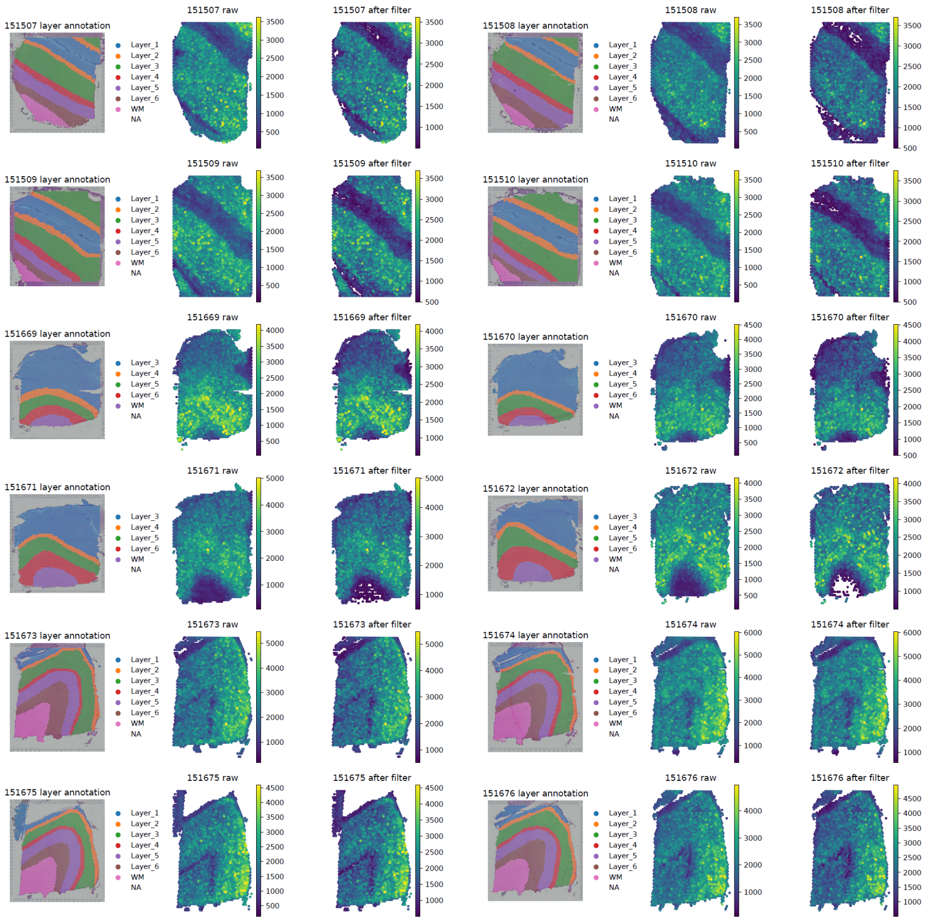

Fig. S1. Filter visualization for the DLPFC dataset of the 10xVisium platform.

a learning rate of 1e-3 and a weight decay of 5e-4 during training. We halted training if the performance on the validation dataset didn't improve over 50 consecutive epochs. We implemented Impeller using PyTorch version 1.12.1 [11] on an Nvidia GeForce RTX 3090 GPU. Our computational infrastructure runs on an AMD EPYC 7662 64-Core Processor with 1.0 TiB memory and uses Ubuntu 20.04.6 LTS system.

In our benchmarking analysis, we employed a variety of existing computational approaches to assess the robustness and accuracy of gene imputation:

1. **scVI and gimVI:** Utilizing the scVI-tools and scVI-external Python packages [5, 17], we implemented these methods. Notably, in our gimVI implementation, the training data encompassed both RNA expression counts and spatial expression information derived from an identical sample, in this man-

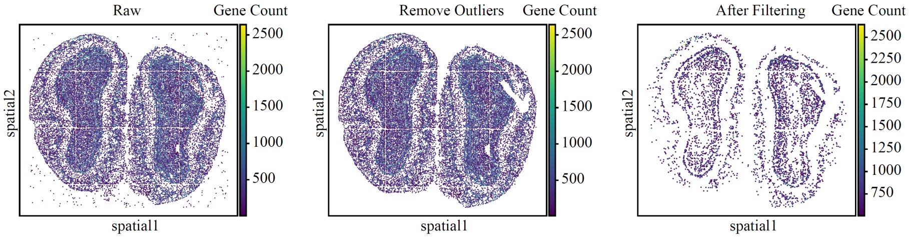

Fig. S2. Filter visualization for the mouse olfactory bulb dataset of the Stereo-seq platform.

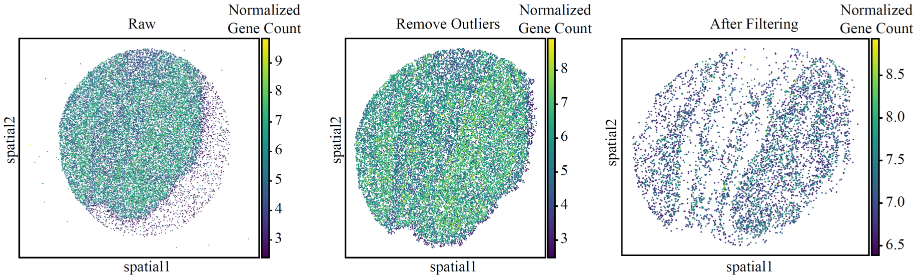

Fig. S3. Filter visualization for the mouse olfactory bulb dataset of the Slide-seqV2 platform.

ner we trained and ran the gimVI model without reference data, creating a reference-free version of the original method. We then employed these methods using their default parameters.

2. **Seurat-based Methods:** Leveraging the Seurat R-package, we employed several strategies to deduce nearest neighbors using either expression data alone or in combination with spatial data [7, 13, 2, 15]. These methods include:
   (a) **Seurat-seKNN (Spatial-Expression-Based K Nearest Neighbor):** For gene imputation, this method combines spatial positioning with gene expression, averaging gene reads from the closest K non-zero neighbors.
   (b) **Seurat-seSNN (Spatial-Expression-Based Shared Nearest Neighbor):** This method computes a weighted average of gene reads, emphasizing shared neighbors.
   (c) **Seurat-eKNN (Expression-Based K Nearest Neighbor):** Focusing only on gene expression, it averages gene reads from the nearest K non-zero neighbors.
   (d) **Seurat-eSNN (Expression-Based Shared Nearest Neighbor):** This method employs a weighted averaging strategy, giving importance to the number of shared neighbors.

   We optimized these KNN networks on the validation dataset to achieve peak performance and subsequently recorded their performance on the test dataset.

Table 1. Gene imputation benchmark for other six samples of the DLPFC dataset. Best resutls are bolded.

Metric		Method	Platform & Dataset					
			10xVisium (DLPFC & Sample ID)					
			151671	151672	151673	151674	151675	151676
L1 Distance	wo	scVI	0.653±0.002	0.673±0.001	0.619±0.004	0.546±0.001	0.696±0.004	0.674±0.003
		ALRA	0.463±0.001	0.469±0.002	0.437±0.005	0.411±0.002	0.460±0.001	0.451±0.002
		eKNN	0.268±0.000	0.271±0.001	0.264±0.001	0.250±0.000	0.269±0.001	0.264±0.000
		eSNN	0.987±0.001	1.019±0.000	0.914±0.001	0.785±0.000	1.059±0.001	1.016±0.001
		Magic	0.652±0.001	0.667±0.001	0.618±0.000	0.544±0.001	0.697±0.000	0.674±0.000
		scGNN	0.519±0.015	0.505±0.011	0.510±0.004	0.450±0.005	0.538±0.009	0.529±0.006
	w	gimVI	0.718±0.001	0.735±0.001	0.673±0.002	0.598±0.000	0.759±0.002	0.735±0.001
		seKNN	0.281±0.000	0.290±0.000	0.283±0.000	0.263±0.000	0.290±0.000	0.283±0.000
		seSNN	0.987±0.001	1.021±0.000	0.915±0.001	0.785±0.000	1.059±0.001	1.015±0.000
		Tangram	1.402±0.001	1.441±0.001	1.297±0.001	1.159±0.000	1.434±0.001	1.399±0.000
		STLearn	1.141±0.001	1.176±0.002	1.030±0.001	0.883±0.001	1.138±0.001	1.109±0.001
		STAGATE	0.277±0.003	0.285±0.004	0.274±0.005	0.256±0.002	0.277±0.000	0.278±0.005
		Impeller	**0.243±0.006**	**0.238±0.002**	**0.239±0.003**	**0.231±0.003**	**0.242±0.006**	**0.233±0.002**
Cosine Similarity	wo	scVI	0.902±0.001	0.903±0.000	0.899±0.001	0.900±0.001	0.899±0.001	0.900±0.001
		ALRA	0.940±0.001	0.940±0.005	0.936±0.008	0.943±0.002	0.946±0.002	0.947±0.003
		eKNN	0.978±0.000	0.979±0.000	0.976±0.000	0.974±0.000	0.979±0.000	0.979±0.000
		eSNN	0.846±0.000	0.849±0.001	0.851±0.000	0.860±0.000	0.841±0.000	0.845±0.001
		Magic	0.907±0.000	0.909±0.000	0.903±0.000	0.904±0.000	0.904±0.000	0.905±0.000
		scGNN	0.923±0.006	0.930±0.003	0.913±0.003	0.916±0.002	0.923±0.002	0.919±0.002
	w	gimVI	0.941±0.001	0.947±0.001	0.939±0.002	0.929±0.000	0.946±0.001	0.944±0.001
		seKNN	0.978±0.000	0.978±0.000	0.975±0.000	0.973±0.000	0.979±0.000	0.978±0.000
		seSNN	0.852±0.000	0.854±0.000	0.855±0.001	0.865±0.000	0.843±0.000	0.848±0.001
		Tangram	0.698±0.000	0.705±0.001	0.699±0.001	0.696±0.001	0.708±0.001	0.701±0.001
		STLearn	0.720±0.001	0.721±0.001	0.728±0.000	0.745±0.001	0.717±0.000	0.722±0.001
		STAGATE	0.978±0.000	0.979±0.000	0.976±0.001	0.973±0.000	0.980±0.000	0.979±0.000
		Impeller	**0.982±0.000**	**0.983±0.000**	**0.981±0.000**	**0.977±0.001**	**0.984±0.000**	**0.983±0.000**
RMSE	wo	scVI	0.783±0.002	0.802±0.001	0.744±0.004	0.657±0.001	0.834±0.005	0.802±0.003
		ALRA	0.725±0.001	0.733±0.005	0.677±0.013	0.622±0.002	0.718±0.001	0.698±0.002
		eKNN	0.366±0.001	0.368±0.001	0.355±0.001	0.331±0.000	0.367±0.002	0.357±0.001
		eSNN	1.111±0.001	1.137±0.000	1.034±0.001	0.894±0.001	1.187±0.001	1.133±0.001
		Magic	0.780±0.001	0.791±0.001	0.742±0.000	0.654±0.000	0.831±0.000	0.799±0.000
		scGNN	0.674±0.023	0.658±0.015	0.667±0.007	0.586±0.007	0.698±0.010	0.693±0.009
	w	gimVI	0.845±0.001	0.852±0.001	0.791±0.002	0.712±0.000	0.881±0.001	0.852±0.001
		seKNN	0.361±0.000	0.372±0.000	0.362±0.000	0.338±0.000	0.372±0.000	0.364±0.000
		seSNN	1.089±0.001	1.115±0.001	1.015±0.001	0.876±0.000	1.164±0.001	1.112±0.001
		Tangram	1.483±0.001	1.520±0.001	1.387±0.001	1.249±0.001	1.523±0.001	1.483±0.001
		STLearn	1.284±0.001	1.316±0.002	1.176±0.000	1.021±0.001	1.310±0.001	1.266±0.001
		STAGATE	0.360±0.004	0.369±0.002	0.355±0.006	0.335±0.002	0.362±0.000	0.361±0.001
		Impeller	**0.326±0.004**	**0.324±0.001**	**0.317±0.002**	**0.309±0.004**	**0.325±0.004**	**0.318±0.001**

3. **ALRA:** For this method, we utilized the Seurat Wrappers package [9]. ALRA is designed to mitigate dropouts by low-rank approximation, offering a unique approach to the gene imputation challenge.
4. **MAGIC:** We implemented this imputation method using the scanpy external package [16]. Similarly to the Seurat-based methods we optimized MAGIC's parameters for the validation dataset before recording its performance on the test dataset.
5. **scGNN:** This method was implemented using the Python package and default parameters [18].
6. **Tangram:** We utilized the Tangram Python package for this implementation [1]. In our approach, we employed the same spatial transcriptomic data for both the scRNA reference and the spatial data, creating a reference-free version of the original method.
7. **stLearn:** This method was implemented using the stLearn Python package and stLearn's default parameters [12].
8. **STAGATE:** This method was implemented using the STAGATE Python package and STAGATE's default parameters [4].

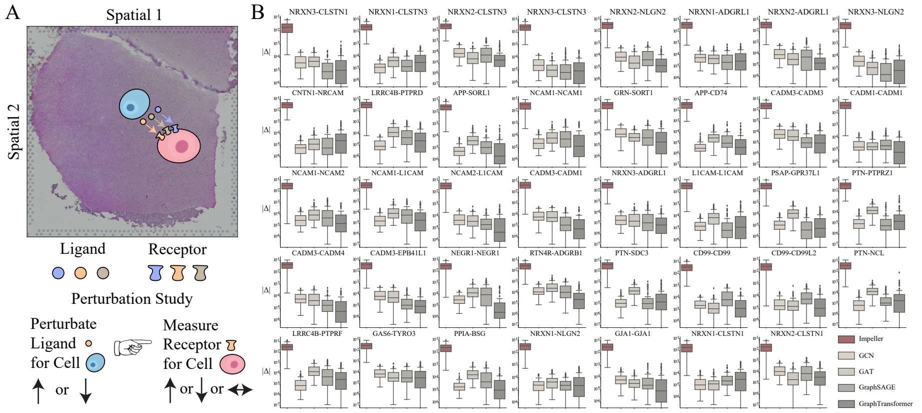

Fig. S4. Perturbation Study. (A): We randomly selected 100 target cells (red) and paired each with a nearby cell (blue) within the same brain layer and a distance of 500 to 600 microns, to simulate long-range CCI. We then altered the ligand gene expression in the blue cells and used various models to assess the receptor gene expression changes in the red cells. (B): Boxplots display the receptor gene expression changes across different ligand-receptor pairs and methods, using a log scale on the y-axis. Results show that Impeller significantly outperforms other graph-based methods, with changes ranging from $10^{-2} \sim 10^{-1}$ compared to $10^{-6} \sim 10^{-4}$, highlighting its superior ability to capture long-range CCI effects.

4 Additional Perturbation Study

We have added an additional perturbation study to demonstrate our model's effectiveness of CCI capture (**Fig. S4**). First, we used the 10X Visium DLPFC dataset (sample 151507) alongside CellChatDB [8], which comprises 3,267 human ligand-receptor (LR) pairs. We identified 39 LR pairs that were highly expressed in this sample, each by over 15% of cells. Then we randomly selected 100 target cells (red cells in **Fig. S4A**) and paired each with a nearby cell (blue cells in **Fig. S4A**) within the same brain layer and spaced 500 to 600 microns apart, simulating long-range CCI. We manipulated the ligand gene expression in the blue cells—expressed genes were set to 0, and unexpressed genes to 10. Using different trained models, we measured the significant changes in receptor gene expression ($|\Delta|$) in the red cells. As shown in **Fig. S4B**, Impeller has significantly larger changes ($10^{-2} \sim 10^{-1}$) than other graph-based methods ($10^{-6} \sim 10^{-4}$), which highlights its superior ability to capture long-range CCI effects.

5 Path Construction Analysis

This section provides an in-depth analysis of different path construction methods and their impacts, focusing on cells at the edges of various layers **Fig. S5A**. We use three path construction methods: our method, derived from Node2Vec

[6]; layer-guided paths, which utilize pre-annotated layer data to ensure paths include only nodes within the same brain layer of the DLPFC; and cell-type-guided paths, which utilize PCA and leiden clustering on gene expression to ensure paths reflect gene similarities by including nodes within the same spatial cluster.

First, we only construct the spatial graph, categorize cells into various groups, and focus on cells at the boundary of layers. The imputed results are shown in **Fig. S6A**. Both the layer-guided and cell-type-guided paths demonstrate enhancements across all cell groups, with notable improvements observed at the border of layer 6 and the white matter (WM), attributed to the distinct gene expression profiles between these layers (**Fig. S7A**). This result indicates that incorporating additional knowledge, such as layer information or gene similarity profiles, into path construction can enhance imputation accuracy when we solely rely on the spatial graph.

Next, we explore the impact of these three path construction methods with both graph modalities (spatial and gene similarity graphs), as shown in **Fig. S6B**. Incorporating gene similarity information, the imputation accuracy of the three methods appears comparable, suggesting that by integrating the gene similarity graph, Impeller can address both spatial influences and gene expression similarities, thus providing effective gene imputation. However, limitations include the absence of pre-annotated layer data in spatial transcriptomics and leiden clustering's resolution sensitivity (**Fig. S7A**), which can markedly impact imputation accuracy with slight adjustments (**Fig. S7B**). We have included optimization options (layer-guided or cell-type-guided spatial path construction) within our framework. With access to reliable annotated layer information and clustering outcomes, Impeller is equipped to utilize these insights for constructing biologically meaningful paths in the spatial graph.

6 Gene Similarity Graph Construction Analysis

This section details our exploration of gene similarity graph construction methods, focusing on the use of highly variable genes (HVG) versus PCA-embedded (20 and 50 dimensional) distances. Our experiments, conducted with the DLPFC dataset, compared the efficacy of these methods in gene imputation accuracy and the corresponding running time. As shown in **Fig. S8A**, PCA slightly increased performance for some samples, such as 151509, 151671, and 151674. Regarding computational effort, we compared the time for HVG/PCA calculations and neighbor searches. As **Fig. S8B** shows, the total time for HVG and 20-PCA is similar. Notably, neighbor search in PCA space is quick. Furthermore, most tasks are completed in under a second, highlighting our graph construction's efficiency.

7 Additional Parameter Analysis

Here we included additional parameter analysis for the number of neighbors in the gene similarity graph. As shown in **Fig. S9**, our method is robust across different numbers of neighbors and samples. We choose 5 as the default number of neighbors to balance the performance and complexity.

References

1. Biancalani, T., Scalia, G., Buffoni, L., Avasthi, R., Lu, Z., Sanger, A., Tokcan, N., Vanderburg, C.R., Segerstolpe, Å., Zhang, M., Avraham-Davidi, I., Vickovic, S., Nitzan, M., Ma, S., Subramanian, A., Lipinski, M., Buenrostro, J., Brown, N.B., Fanelli, D., Zhuang, X., Macosko, E.Z., Regev, A.: Deep learning and alignment of spatially resolved single-cell transcriptomes with tangram. Nature Methods **18**(11), 1352–1362 (Nov 2021). https://doi.org/10.1038/s41592-021-01264-7
2. Butler, A., Hoffman, P., Smibert, P., Papalexi, E., Satija, R.: Integrating single-cell transcriptomic data across different conditions, technologies, and species. Nature Biotechnology **36**, 411–420 (2018). https://doi.org/10.1038/nbt.4096
3. Chen, A., Liao, S., Cheng, M., Ma, K., Wu, L., Lai, Y., Yang, J., Li, W., Xu, J., Hao, S., et al.: Large field of view-spatially resolved transcriptomics at nanoscale resolution. BioRxiv **2021** (2021)
4. Dong, K., Zhang, S.: Deciphering spatial domains from spatially resolved transcriptomics with an adaptive graph attention auto-encoder. Nature communications **13**(1), 1739 (2022)
5. Gayoso, A., Lopez, R., Xing, G., Boyeau, P., Valiollah Pour Amiri, V., Hong, J., Wu, K., Jayasuriya, M., Mehlman, E., Langevin, M., Liu, Y., Samaran, J., Misrachi, G., Nazaret, A., Clivio, O., Xu, C., Ashuach, T., Gabitto, M., Lotfollahi, M., Svensson, V., da Veiga Beltrame, E., Kleshchevnikov, V., Talavera-López, C., Pachter, L., Theis, F.J., Streets, A., Jordan, M.I., Regier, J., Yosef, N.: A python library for probabilistic analysis of single-cell omics data. Nature Biotechnology **40**(2), 163–166 (Feb 2022). https://doi.org/10.1038/s41587-021-01206-w
6. Grover, A., Leskovec, J.: node2vec: Scalable feature learning for networks. In: Proceedings of the 22nd ACM SIGKDD international conference on Knowledge discovery and data mining. pp. 855–864 (2016)
7. Hao, Y., Hao, S., Andersen-Nissen, E., III, W.M.M., Zheng, S., Butler, A., Lee, M.J., Wilk, A.J., Darby, C., Zagar, M., Hoffman, P., Stoeckius, M., Papalexi, E., Mimitou, E.P., Jain, J., Srivastava, A., Stuart, T., Fleming, L.B., Yeung, B., Rogers, A.J., McElrath, J.M., Blish, C.A., Gottardo, R., Smibert, P., Satija, R.: Integrated analysis of multimodal single-cell data. Cell (2021). https://doi.org/10.1016/j.cell.2021.04.048
8. Jin, S., Guerrero-Juarez, C.F., Zhang, L., Chang, I., Ramos, R., Kuan, C.H., Myung, P., Plikus, M.V., Nie, Q.: Inference and analysis of cell-cell communication using cellchat. Nature communications **12**(1), 1088 (2021)
9. Linderman, G.C., Zhao, J., Kluger, Y.: Zero-preserving imputation of scrna-seq data using low-rank approximation. bioRxiv (2018). https://doi.org/10.1101/397588
10. Maynard, K.R., Collado-Torres, L., Weber, L.M., Uytingco, C., Barry, B.K., Williams, S.R., Catallini, J.L., Tran, M.N., Besich, Z., Tippani, M., et al.: Transcriptome-scale spatial gene expression in the human dorsolateral prefrontal cortex. Nature neuroscience **24**(3), 425–436 (2021)

11. Paszke, A., Gross, S., Massa, F., Lerer, A., Bradbury, J., Chanan, G., Killeen, T., Lin, Z., Gimelshein, N., Antiga, L., et al.: Pytorch: An imperative style, high-performance deep learning library. Advances in neural information processing systems **32** (2019)
12. Pham, D., Tan, X., Xu, J., Grice, L.F., Lam, P.Y., Raghubar, A., Vukovic, J., Ruitenberg, M.J., Nguyen, Q.: stlearn: integrating spatial location, tissue morphology and gene expression to find cell types, cell-cell interactions and spatial trajectories within undissociated tissues. bioRxiv (2020). https://doi.org/10.1101/2020.05.31.125658
13. Satija, R., Farrell, J.A., Gennert, D., Schier, A.F., Regev, A.: Spatial reconstruction of single-cell gene expression data. Nature Biotechnology **33**, 495–502 (2015). https://doi.org/10.1038/nbt.3192
14. Stickels, R.R., Murray, E., Kumar, P., Li, J., Marshall, J.L., Di Bella, D.J., Arlotta, P., Macosko, E.Z., Chen, F.: Highly sensitive spatial transcriptomics at near-cellular resolution with slide-seqv2. Nature biotechnology **39**(3), 313–319 (2021)
15. Stuart, T., Butler, A., Hoffman, P., Hafemeister, C., Papalexi, E., III, W.M.M., Hao, Y., Stoeckius, M., Smibert, P., Satija, R.: Comprehensive integration of single-cell data. Cell **177**, 1888–1902 (2019). https://doi.org/10.1016/j.cell.2019.05.031
16. van Dijk, D., Sharma, R., Nainys, J., Yim, K., Kathail, P., Carr, A.J., Burdziak, C., Moon, K.R., Chaffer, C.L., Pattabiraman, D., Bierie, B., Mazutis, L., Wolf, G., Krishnaswamy, S., Pe'er, D.: Recovering gene interactions from single-cell data using data diffusion. Cell **174**(3), 716–729.e27 (2018). https://doi.org/https://doi.org/10.1016/j.cell.2018.05.061
17. Virshup, I., Bredikhin, D., Heumos, L., Palla, G., Sturm, G., Gayoso, A., Kats, I., Koutrouli, M., Angerer, P., Bergen, V., Boyeau, P., Büttner, M., Eraslan, G., Fischer, D., Frank, M., Hong, J., Klein, M., Lange, M., Lopez, R., Lotfollahi, M., Luecken, M.D., Ramirez, F., Regier, J., Rybakov, S., Schaar, A.C., Valiollah Pour Amiri, V., Weiler, P., Xing, G., Berger, B., Pe'er, D., Regev, A., Teichmann, S.A., Finotello, F., Wolf, F.A., Yosef, N., Stegle, O., Theis, F.J., Community, S.: The scverse project provides a computational ecosystem for single-cell omics data analysis. Nature Biotechnology **41**(5), 604–606 (May 2023). https://doi.org/10.1038/s41587-023-01733-8
18. Wang, J., Ma, A., Chang, Y., Gong, J., Jiang, Y., Qi, R., Wang, C., Fu, H., Ma, Q., Xu, D.: scgnn is a novel graph neural network framework for single-cell rna-seq analyses. Nature communications **12**(1), 1882 (2021)

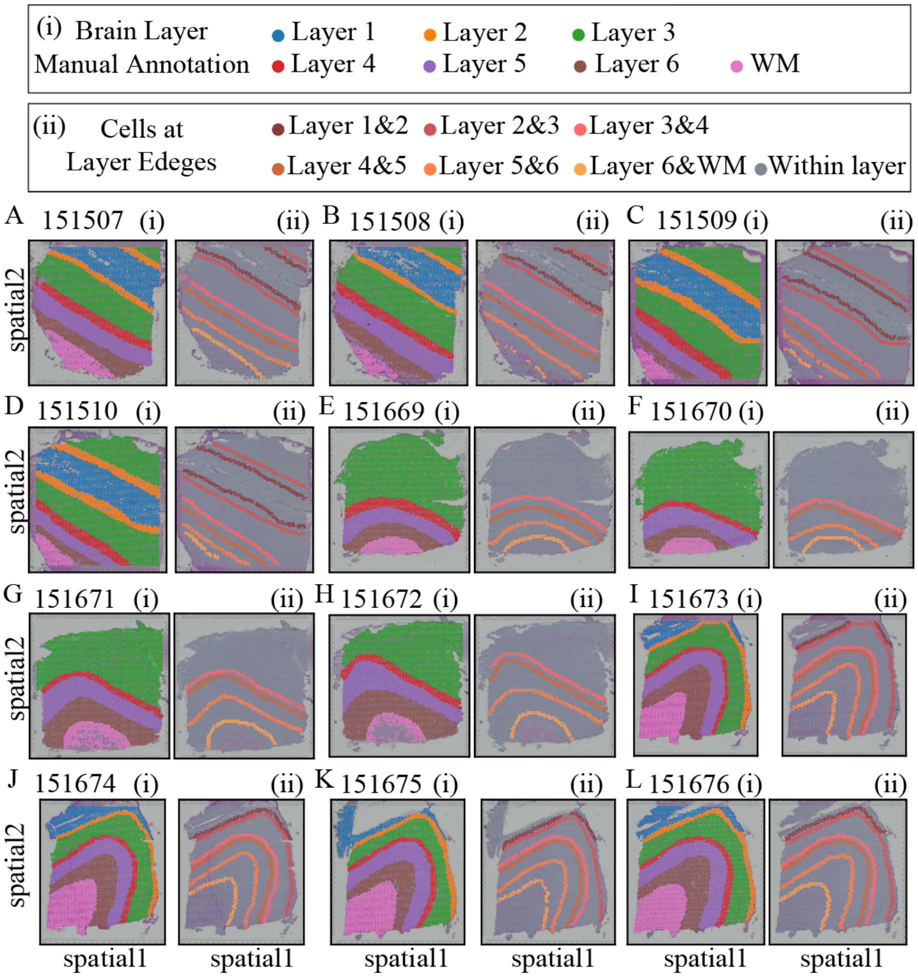

Fig. S5. DLPFC dataset's 12 sample border cell visualizations.

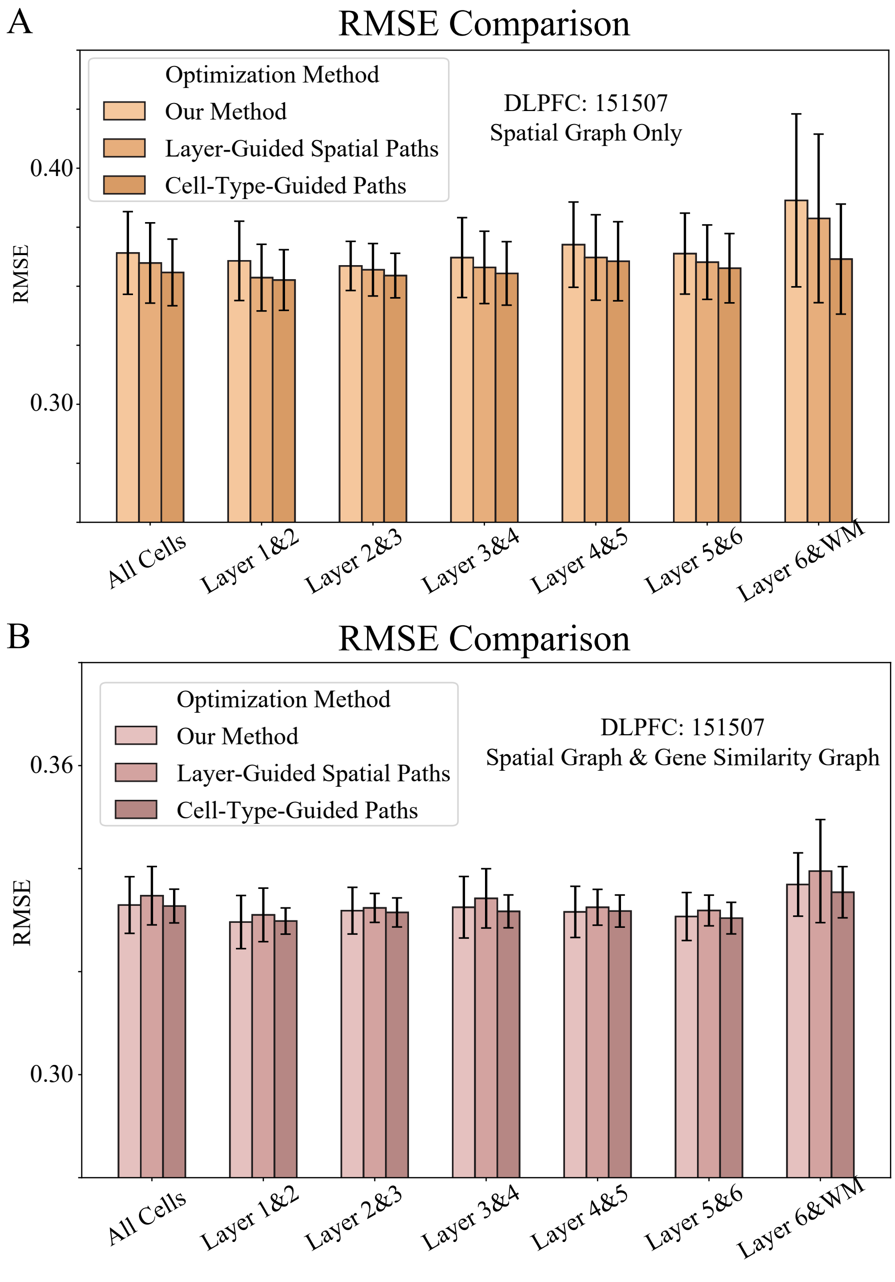

Fig. S6. Path construction comparison. (A) Comparison for all cells. (B) Comparison for border cells.

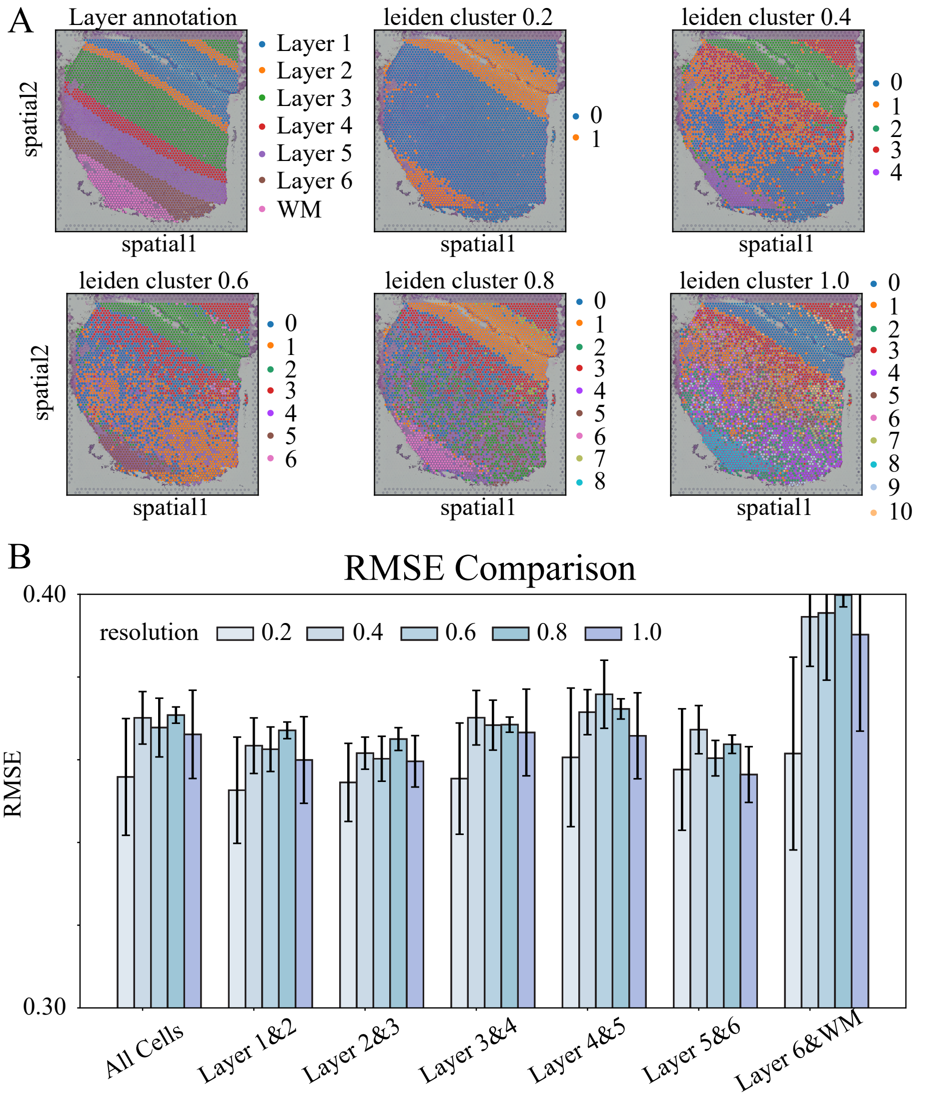

Fig. S7. Path construction comparison. (A) Comparison for all cells. (B) Comparison for border cells.

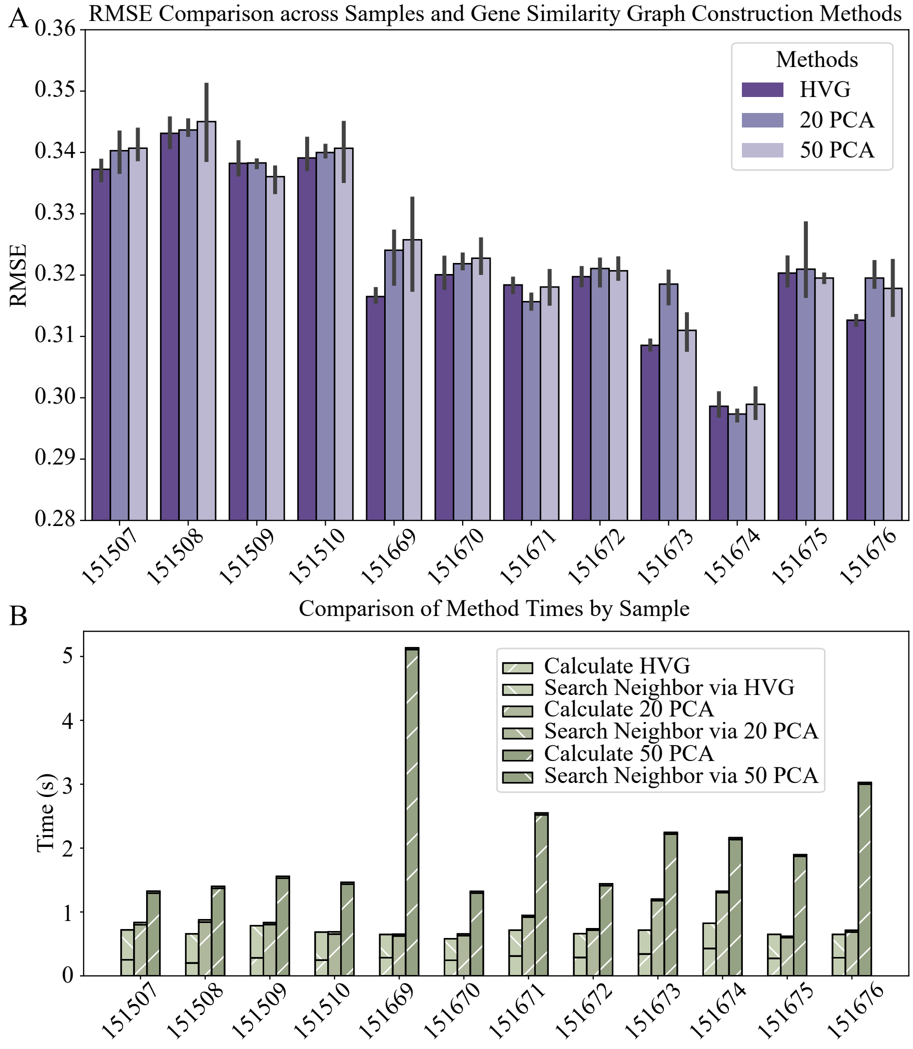

Fig. S8. Gene similarity graph construction comparison.

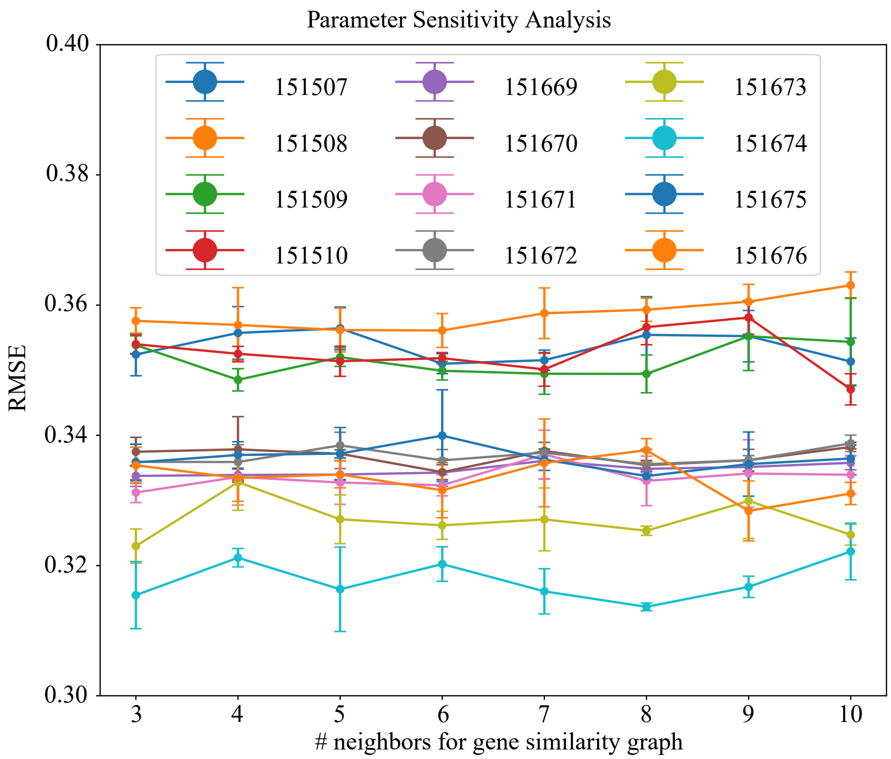

Fig. S9. Gene similarity graph construction comparison.
